# Supplementary material for: Network assessment and modeling the management of an epidemic on a college campus with testing, contact tracing, and masking
Source: PLoS One. 2021 Sep 17;16(9):e0257052. doi: 10.1371/journal.pone.0257052 (PMC8448338; doi:10.1371/journal.pone.0257052)
Supplement: Attachment — Submitted filename: Response-to-Reviewers.pdf [file pone.0257052.s001.pdf]

July 30, 2021

PLoS ONE

Dear PLoS Editor(s)

Thank you for the opportunity to revise this manuscript. I am grateful to you and the two reviewers for your helpful comments. I think the paper is much improved and of interest to the readers of PLoS ONE!

I have submitted two versions of the manuscript. One includes the changes tracked in LaTeX. New text is in red and removed text is marked with strike-out lines (e.g., ~~remove this~~). A clean copy with these changes also is included.

Below are items raised in the reviews and how I addressed them.

1. More emphasis was added regarding the importance of studying highly-transmissible diseases specifically on college and university campuses. In particular, the motivation for this study, further explained in the paper, is to explore practices that increase the safety of the millions of young adults in college and university settings. There are nearly 2700 four-year institutions in the United States, for instance, which have approximately 10 million students (National Center for Education Statistics).
2. I removed the use of first person narrative throughout.
3. I appreciate Reviewer #1's comment that "this paper seems a little untimely" since "the mask mandate for individuals who are vaccinated is no longer applicable." In the paper's defense, just this week the CDC recommended vaccinated people return to wearing masks indoors. Additionally, the FDA has not formally approved the vaccine so most institutions are unable to require students to get vaccinated. Therefore, masks and the process of testing, isolating infections individuals and quarantining contacts remain important and effective strategies to reduce transmission. This paper explores these in a real-world setting. Additionally, colleges and universities are beginning another academic year with great uncertainly about the risk of COVID-2 in academic communities, particularly with the rise of more highly transmissible variants in the population. Finally, there certainly will be new viral pandemics in our future and colleges and universities, with millions of students in the USA alone, need to understand how such epidemics can spread and be controlled.
4. Reviewer #1 suggested the model could include vaccination. This is a great idea but would greatly extend this paper and complicate the results because, undoubtedly, the additional factors (vaccination rates, vaccination strategies, and a different vaccine efficacies) would interact with the five factors discussed in this paper, creating an eight factor analysis. [As an aside, in the coming weeks I will be submitting a vaccination paper and then another on accounting for the evolution of SARS-CoV-2 variants in more general settings.]
5. Reviewer #1 suggested that hand washing and social distancing might be included. Research does not support that hand-washing significantly reduces the spread of this airborne virus and was, therefore, not considered. Social distancing is difficult to control and the actual distances needed are not well understood. In contrast, the data on mask efficacies and rates at which people comply in wearing masks are well understood.
6. Reviewer #1 suggests including a diagram of the layout of the residential housing. I think this might incorrectly imply that the model incorporates this level of detail. The model makes no assumptions about distances between residence halls and classrooms or among student rooms and other rooms in the same or different hallways or halls. It is assumed that the primary opportunities for viral transmission occur inside classrooms and between students living together. The model is constrained by existing data as represented in Figure 1.
7. Reviewer #1 asked for the number of students with the percentage mentioned (89% of the 5539 students) so I added this and referred to the percentage parenthetically.

8. Reviewer #1 suggested that the "five factors" be reiterated. I did this by directing the reader to Table 2.
9. Reviewer #1 suggested that I clarify what the "higher-order interactions" are. This was added.
10. Reviewer #1 states they are confused by testing daily. This is one of the factors (testing cycles ranged from none, once per semester, up to every day). To clarify this I added a reference to Figure 9 which shows the different frequencies of testing and how it affects the total number of tests administered.
11. Review #2 requested clarification for the origin of parameters and so a reference to Table 2, which contains these parameter sources, was added at the first mention of using R_0.
12. Greater detail and justification for why the SUNY Geneseo network was used has been added. Also, additionally information on the size of the institution was included. Geneseo is a good model for this study because it is a relatively self-contained, rural, residential, undergraduate institution. There are several dozen graduate students that enroll in their own courses or have placements and residences off campus (there is no on campus housing for graduate students).
13. Faculty and staff are not included in the model. It is assumed, and is the focus of this study, that students interact primarily with each other, including in classrooms and in residence halls. This is discussed in the new limitations paragraph.
14. Reviewer #2 asked for additional information on why this school was chosen and to provide additional background information. This has been added to the Methods section.
15. For clarification I changed the model acronym from "SEIRIQ" to "$SEIRI_{sol}Q$" to reduce confusion that might arise from having two "I" states.
16. Reviewer #2 suggested the figure comparing college and random housing networks could be improved by reducing the number of vertices. This is a great suggestion and was done with a smaller subset of students. The figure caption was updated accordingly.
17. Reviewer #2 reiterates the need for clarification of purpose and limitations, which are now more complete in the manuscript.
18. I have added a paragraph at the end of the Discussion on limitations. I think these better help the reader interpret the results of the paper.

Thank you again for your consideration of this manuscript.

Sincerely

Gregg Hartvigsen
Biology Department
SUNY Geneseo
Geneseo, NY 14454
hartvig@geneseo.edu
